# Reflections and progress in conservation physiology

**DOI:** 10.1093/conphys/cow071

**Published:** 2017-01-04

**Authors:** Steven J. Cooke, Kevin R. Hultine, Jodie L. Rummer, Craig E. Franklin

**Affiliations:** 1 Fish Ecology and Conservation Physiology Laboratory, Department of Biology and Institute of Environmental Science, Carleton University, 1125 Colonel By Drive, Ottawa, ON,CanadaK1S 5B6; 2 Department of Research, Conservation and Collections, Desert Botanical Garden, 1201 North Galvin Parkway, Phoenix, AZ 85008, USA; 3 ARC Centre for Excellence for Coral Reef Studies, James Cook University, Townsville, QLD 4811, Australia; 4 School of Biological Sciences, The University of Queensland, Brisbane, QLD 4072, Australia

As the journal *Conservation Physiology* enters its fifth year of publication, we reflect on where we have come from and look forward to where we are going. There has been much progress in the emerging discipline of conservation physiology, and more than 200 papers have been published by the journal, including many of the most exciting developments. The journey began with a refined definition of conservation physiology (Cooke *et al*., 2013) and quickly moved forward with the development of a conceptual framework for the discipline (Coristine *et al*., 2014).

One of our key aims is to link mechanisms, policy and practice, and it is therefore appropriate that the journal has become the natural home for papers that are expanding and validating the ‘conservation physiology toolbox’. Some of the highlights include developments in endocrinology (e.g. measuring glucocorticoids in feathers; Berk *et al*., 2016), point-of-care devices (e.g. assessing physiological status of alligators; Hamilton *et al*., 2016) and colour-based animal biomarkers (e.g. as indicators of exposure to pollutants; Lifshitz and St Clair, 2016). The conservation physiology toolbox is also expanding in plant research, including approaches focused on non-structural carbohydrate storage (e.g. as indicators of plant tolerance to herbivory; Vilela *et al*., 2016). These papers are providing a real service to the conservation community and enable non-invasive, empirical physiological studies on rare or threatened taxa.

Conservation physiology is identifying cause-and-effect relationships (Tracy *et al*., 2006), and there are now many examples where the mechanisms underlying or potentially contributing to population declines have been elucidated. For example, Hancock and Place (2016) studied the hypoxia tolerance of woolly sculpin and revealed that these fish may be exposed to conditions near their physiological limits if faced with future ocean acidification projections. Carroll *et al*. (2016) revealed that little penguins showed evidence of sensitization rather than habituation to human-caused disturbance. In some cases, however, efforts to assess the consequences of apparent stressors or disturbances on various organisms produced equally informative results indicating a lack of any negative physiological consequences (e.g. no effects of human activity on the stress response in painted turtles; Polich, 2016). Of late, there has also been interest in evaluating biomarkers that could serve as predictors of population health and status. Interestingly, Madliger and Love (2016) revealed that stress hormones measured on a subset of individuals may not provide clarity as to how entire populations respond to environmental change. Findings by Madliger and Love have been further supported with results from Killen *et al*. (2016), where the need to sample individuals through time given inherent repeatability of many physiological metrics was demonstrated (Killen *et al*., 2016).

Although conservation physiology is certainly effective at identifying problems, there are also a growing number of success stories (see Madliger *et al*., 2016) where physiological concepts, knowledge and tools have helped to shape policy and management actions. Thus, physiological knowledge can inform development of policy options for management of marine fishes (McKenzie *et al*., 2016) including, for example, Pacific salmon (Patterson *et al*., 2016) and pelagic fisheries (Horodysky *et al*., 2016). Potential applications were also the core theme of the highly successful ‘Conservation Physiology’ themed 3 day symposium at the Society for Experimental Biology conference held in Brighton, UK last summer. Look out for papers from each of the keynote speakers appearing in the journal over the next few months.

Despite these ‘successes’, this does not mean that it is time to celebrate, because many populations of wild organisms continue to decline as a consequence of expanding human enterprise across the globe. Indeed, today more than ever, there is a need for mission-oriented fields of study, such as conservation physiology. We hope that the journal will continue to shape how the field develops such that those working in the realm of conservation physiology have the potential to be influential and even transformative with their science, practice and communication. In some cases, it will be meta-analysis and structured literature reviews that help to codify scientific knowledge, which is something we are starting to see occur in conservation physiology (e.g. Lefevre, 2016). Two special issues in 2016, one focused on conservation physiology of migration (Lennox *et al*., 2016) and the other on conservation physiology of marine fishes (McKenzie *et al*., 2016), emphasized the community's collective interest around these topics, both of which have extensive policy and management links.

The journal itself has continued to evolve with the direction from a talented and dedicated editorial board, and there are exciting developments on the horizon to share with the community. First and foremost, we are pleased to announce that, in 2017, we will receive our first Impact Factor from ISI Thompson-Reuters. In 2016, we were formally indexed in Web of Knowledge, which now includes all content back to 2013, and full content is also indexed in Scopus and PubMed Central. These achievements are a testament to the quality of content and the influence that the work that authors share with us is having on the scientific community. However, Impact Factor alone is only a part of the story. We are equally excited about the attention that our papers are receiving from the public, traditional media, and social media, as monitored by Altmetrics. Many of our papers are being discussed and shared on platforms such as Facebook and Twitter, and in 2016, we started a Twitter account for the journal (managed by Jacqueline Chapman; @conphysjournal).

In 2017, we will launch two new types of articles, both of which are intended to engage our community further. Conservation Physiology in Action (CPiA) will be short (~500 word) and engaging summaries written by early career researchers under the guidance of CPiA Editor Jodie Rummer. The idea behind CPiA is to share exciting content in a manner that is accessible and interesting to the broader community, including end users of the information. Voices in Conservation Physiology (ViCP) will be a series of short (~2000 word) essays from prominent members of the conservation physiology community who have been working in this realm for most of their careers (even before it was called conservation physiology!). Contributors will provide their perspective on the past, present and future of conservation physiology related to their area of expertise. The ViCP contributions will include some level of synthesis about their career, while making connections to the broader literature/field/discipline. Keep your eye open for these exciting additions to the journal!

Mark Van Kleunen will step down as Plant Science Editor at the end of 2016, and we wish to thank him for his outstanding contributions and leadership. At the same time, we are pleased to announce that Kevin Hultine from the Desert Botanical Garden in Arizona joins us as the new Plant Science Editor.

As we launch our fifth year, we also want to let all of our contributors and readers know that we are keen to hear from you. Do not hesitate to reach out to any member of the editorial team with ideas for content, special issues, to participate in CPiA or ViCP, or for anything else that you think would help to make the journal better. This is YOUR journal!
